# The income-based disparities in preeclampsia and postpartum hemorrhage: a study of the Korean National Health Insurance cohort data from 2002 to 2013

**DOI:** 10.1186/s40064-016-2620-8

**Published:** 2016-06-27

**Authors:** Seung-Ah Choe, Hye-Sook Min, Sung-Il Cho

**Affiliations:** Department of Obstetrics and Gynecology, CHA Gangnam Medical Center, CHA University, Seoul, Republic of Korea; Department of Preventive Medicine, Graduate School of Public Health, Seoul National University, Seoul, Republic of Korea; Department of Epidemiology, Graduate School of Public Health and Institute of Health and Environment, Seoul National University, 1 Gwanak-ro, Gwanak-gu, Seoul, 151-742 Republic of Korea

**Keywords:** Disparity, Preeclampsia, Korean National Health Insurance

## Abstract

There is limited evidence on the effects of relatively low socioeconomic status on maternal health. Additionally, the global economic recession that began in 2008 could have worsened disparities in maternal complications. To explore disparities in maternal health, we analyzed the occurrence of preeclampsia and postpartum hemorrhage according to level of household income. A population-based cohort data set from the Korean National Health Insurance was used to calculate the age-adjusted incidence, slope index of inequality, and Kunst and Mackenbach relative index of inequality (RII_KM_) for preeclampsia and postpartum hemorrhage from 2002 to 2013. In the aggregated data of 65,479 live births, women with lower household income showed a higher risk of developing preeclampsia and postpartum hemorrhage than those with higherhigher incomes after adjusting for conventional risk factors. The absolute and relative inequalities for both complications showed no significant change over the period from 2002 to 2013. Considering the difference in the trends and risks of major obstetric complications according to level of household income, policies to monitor and reduce disparities in maternal health across different economic levels need to be implemented.

## Background

Several lines of evidence indicate that socioeconomic disadvantages are related to maternal complications during or after childbirth (Lindquist et al. 2013, 2014). The risk of perinatal adverse outcomes, such as birth complications, lower birth weight, and preterm or early term birth are higher in lower income groups than in higher income groups (Starfield et al. 1991; Borders et al. 2007). Even in developed countries, lower socioeconomic status may pose additional risks for poorer maternal health (Lee et al. 2005). Women in manual occupations are more likely to experience severe morbidities during pregnancy and labor compared with those in managerial/professional jobs. This socioeconomic gradient of severe maternal morbidities is independent of ethnicity, body mass index (BMI), and age (Lindquist et al. 2013). In a Finnish study, mothers of lower socioeconomic status were more likely to undergo Cesarean deliveries than those of higher status (Raisanen et al. 2014a). Living in a disadvantaged area is also an independent risk factor for poorer maternal health outcomes, as shown in an Australian study (Lindquist et al. 2014).

Maternal complications during delivery are not expected routinely in the delivery process but adversely affect the mother’s physical health during and after childbirth. The prevalence of any type of maternal complication during labor and delivery ranges between 13 % (Say et al. 2004) and 43 % (Danel et al. 2003). Among maternal complications, preeclampsia and postpartum hemorrhage are the most common causes of maternal mortality worldwide. In countries with relatively lower incidences of maternal mortality, the most common complications are preeclampsia (and/or eclampsia), gestational diabetes mellitus, postpartum hemorrhage, and bacterial sepsis (Berg et al. 2009; Danel et al. 2003; Nair et al. 2015). Preeclampsia is a multi-organ disorder that occurs in 2–8 % of pregnancies beyond 20 weeks of gestation (Duley 2009). Affected mothers are at risk of emergency operative deliveries and subsequent mortality and morbidities, including postpartum hemorrhage (Gibbons et al. 2014; Chauhan et al. 2014). With the projected increase in mothers’ ages, the burden of these conditions is expected to rise (Cavazos-Rehg et al. 2014).

Many researches have indicated that the health status of disadvantaged members of society could have worsened with the recent global economic recession, thereby exacerbating health disparities (Bacigalupe and Escolar-Pujolar 2014). Likewise, economic depressions at the national level could have widened the income-based gap in maternal health. Beyond the identification of health disparities, monitoring the temporal changes in disparities is also important for developing interventions to reduce it (Khang et al. 2008; Savard et al. 2013). However, limited evidence is available regarding disparities in maternal health along the socioeconomic gradient. To identify and examine the temporal changes in disparities in preeclampsia and postpartum hemorrhage during the past decade, we explored the obstetric delivery data in the Korean National Health Insurance (NHI) cohort database.

## Methods

We used a population-based cohort dataset from the NHI, which covers >95 % of all Korean nationals residing in Korea since 1989 (Kwon 2003). The NHI database contains all the information on the diagnoses, prescribed medications, procedures, and treatments covered by NHI. The present cohort consisted of NHI data, from 2002 to 2013, of around a million individuals obtained by a stratified random sampling method according to gender, age group, and income level. The representativeness of this cohort data set was confirmed in the study of Lee et al. (Lee 2014). The individual data in the cohort is anonymized to comply with the Personal Information Protection Act. Because limited information is included on individual institutions, each institution cannot be identified separately within the cohort dataset.

The validity of the Korean NHI database, which has been reported to be acceptable in several investigations, has been used in numerous epidemiological studies (Cho et al. 2013, 2015; Lee 2014; Kim et al. 2012). Although the accuracy of diagnostic coding could vary substantially across conditions (Fisher et al. 1992), the codes for more severe conditions have been reported to be more accurate than those for less severe ones (Park et al. 2003). Because there is no previous study on the validity of obstetric complications in the Korean NHI cohort data, working definitions using procedure codes specific to the conditions were used in this study. We identified individuals with obstetric delivery discharge from the NHI cohort data according to three criteria, following the hierarchical method suggested by Kuklina et al. (2008): (1) cases with a treatment history in an obstetrics and gynecology department and with a diagnostic code for delivery (starting with ‘O8’ in International Statistical Classification of Disease and Related Health Problems, 10th Revision [ICD-10]); (2) cases with a hospital admission made under a delivery code, and (3) cases aged between 15 and 44 years. The presence of preeclampsia and postpartum hemorrhage during labor or delivery was determined by coexistence of diagnostic and specific procedure codes for both complications within the obstetric delivery data. For preeclampsia, the ICD-10 codes for preeclampsia (O14.0, mild-to-moderate pre-eclampsia, O14.1, severe pre-eclampsia, O14.2, HELLP (hemolysis, elevated liver enzymes, and lower platelets) syndrome, and O14.9, pre-eclampsia, unspecified) and the procedure code for intravenous MgSO_4_, which is used in severe cases of preeclampsia, was used in the identification of preeclampsia cases. For postpartum hemorrhage, ICD-10 codes starting with O72.0–O72.3, and procedure codes for blood transfusions, were included in the working definition.

The Korean NHI cohort database includes individually-linked data of household income decile. The household income levels of individual data were divided into three groups (lower: 10–40 %, middle: 40–80 %, and higher: 80–100 %) for analytical convenience. The incidences of preeclampsia and postpartum hemorrhage in each income group were estimated using regression models considering the mother’s age in the corresponding year. The analyses were performed using SAS software (ver. 9.3; SAS Institute Inc., Cary, NC, USA).

To identify changes in incidences and inequalities over time, the study period was divided into three portions (2002–2005, 2006–2009, and 2010–2013). In each period, age-adjusted incidences in the three income groups were calculated. The income-based inequality was measured by the slope index of inequality (SII) and the Kunst and Mackenbach relative index of inequality (RII_KM_), which are widely used indices in social epidemiology. Unlike traditional indicators, such as risk ratios and risk differences, the SII and RII_KM_ consider the distribution of socioeconomic advantage for the target population. As an absolute scale of disparity, SII can be interpreted as the difference between the bottom and top of the social group hierarchy. For a relative disparity scale, the RII_KM_ is estimated by dividing the health status of the least advantaged by that of the most advantaged, in terms of the social group hierarchy (Mackenbach and Kunst 1995). Using the highest social rank as a reference, an RII_KM_ value >1 indicates that the morbidity rate is higher among groups with lower social ranks (Avendano et al. 2010). Compared with traditional relative risk measures, these scales use data for all social groups and account for group size (Harper and Lynch 2005). The SII and RII_KM_, and their 95 % confidence intervals (CIs), were calculated using the Health Disparities Calculator (HD*Calc, ver. 1.2.4; October 29, 2013; Division of Cancer Control and Population Sciences, Surveillance Research Program and Applied Research Program, National Cancer Institute). Plotting of these inequality measures in the three periods was done with the ‘R’ software (ver. 3.0.3; R Development Core Team, Vienna, Austria). This study was reviewed and approved by the Seoul National University Institutional Review Board (IRB No. 1412/001-010).

## Results

In total, the number of cases with obstetric delivery discharges in the Korean NHI database from 2002 to 2013 was 65,479. The clinical characteristics and outcomes among the three income groups are summarized in Table 1. During the study period, younger age, being in paid work, and nulliparity were more prevalent in the lower income group than in the others. The proportion of multiple gestations (1.76 % in higher, 1.12 % in middle, and 1.05 % in lower income group) and diabetes (1.98 % in higher, 1.38 % in middle, and 1.33 % in lower income group) were highest in the higher income group. Among the two obstetric complications, postpartum hemorrhage occurred more frequently in women with lower and middle household income in the analysis of the aggregated data.

**Table 1:** Household income-based differences in the clinical characteristics and obstetric outcomes in live birth cases in the Korean National Health Insurance cohort database, from 2002 to 2013 (aggregated data)

Variables	Household income level			
	Lower (N = 27,258)	Middle (N = 20,066)	Upper (N = 18,155)	P value (Df = 1)^a^
	Number (%)	Number (%)	Number (%)	
*Age group*				
15–19 years	165 (1.01)	85 (0.29)	43 (0.21)	<.001
20–24 years	1733 (10.66)	1453 (5)	602 (2.9)	
25–29 years	6592 (40.55)	11,786 (40.57)	5888 (28.4)	
30–34 years	5743 (35.33)	12,528 (43.12)	10,278 (49.58)	
35–39 years	1683 (10.35)	2784 (9.58)	3400 (16.4)	
40–44 years	341 (2.1)	417 (1.44)	520 (2.51)	
Worker	4411 (27.13)	7417 (25.53)	3877 (18.7)	<.001
*Obstetric characteristics* ^b^				
Nulliparity	10,543 (64.85)	18,882 (64.99)	12,135 (58.54)	<.001
Multiple gestation	171 (1.05)	325 (1.12)	364 (1.76)	<.001
Diabetes	217 (1.33)	400 (1.38)	411 (1.98)	<.001
Induction of labor	2933 (18.04)	5485 (18.88)	3905 (18.84)	0.066
Cesarean delivery	6157 (37.87)	10,680 (36.76)	7593 (36.63)	0.018
*Outcomes*				
Preeclampsia treated with MgSO_4_	153 (0.94)	235 (0.81)	174 (0.84)	0.332
Postpartum hemorrhage requiring blood transfusion	420 (2.58)	701 (2.41)	469 (2.26)	0.046

Values are presented as number (percentages in parentheses)

^a^The Mantel–Haenszel Chi square tests were done for exploring a linear association between household income groups

^b^Informations on nulliparity, multiple gestation, induction of labor and cesarean delivery were available from treatment code in the Korean NHI database

Table 2 shows the adjusted odds ratios (ORs) of developing preeclampsia and postpartum hemorrhage, from the multivariable models which included age group, being a paid worker, income level, nulliparity, multiple gestation, diabetes, Cesarean delivery and induction of labor (the last two variables are included only in the model for postpartum hemorrhage). The lower household income group showed an independently increased risk for preeclampsia (OR 1.26, 95 % CI 1.01–1.57) and postpartum hemorrhage (OR 1.21, 95 % CI 1.06–1.39), compared with the higher income group. Women who were paid workers at the time of pregnancy were less likely to experience postpartum hemorrhage than those who were not (OR 0.87, 95 % CI 0.77–0.99). Among the clinical factors, maternal age ≥35 years, multiple gestation, and presence of diabetes increased the risk for developing preeclampsia. For postpartum hemorrhage, maternal age <20 or ≥35 years, multiple gestation, presence of diabetes, Cesarean delivery, and induction of labor were risk factors.

**Table 2:** Adjusted odds ratios of the clinical characteristics for preeclampsia and postpartum hemorrhage in live birth cases in the Korean National Health Insurance cohort database, from 2002 to 2013 (aggregated data)

	Odds ratio (95 % confidence intervals)^a^	
	Preeclampsia	Postpartum hemorrhage
*Age group*		
15–19 versus 25–29	0.96 (0.24, 3.91)	2.99 (1.78, 5.01)
20–24 versus 25–29	0.86 (0.56, 1.33)	1.09 (0.86, 1.38)
30–34 versus 25–29	1.05 (0.86, 1.29)	1.11 (0.99, 1.25)
35–39 versus 25–29	1.43 (1.11, 1.85)	1.34 (1.15, 1.57)
40–44 versus 25–29	1.90 (1.20, 3.00)	1.88 (1.43, 2.47)
Worker	0.99 (0.81, 1.21)	0.87 (0.77, 0.99)
*Income groups*		
Lower versus Upper	1.26 (1.01, 1.57)	1.21 (1.06, 1.39)
Middle versus Upper	1.08 (0.88, 1.32)	1.15 (1.02, 1.30)
Nulliparity	0.77 (0.65, 0.92)	0.85 (0.76, 0.94)
Multiple gestation	6.17 (4.44, 8.59)	2.87 (2.23, 3.70)
Diabetes	3.83 (2.69, 5.43)	1.97 (1.49, 2.60)
Cesarean delivery	–	2.05 (1.82, 2.30)
Induction of labor	–	1.20 (1.03, 1.41)

^a^With adjustment for every other variable in the model

**Fig. 1:**
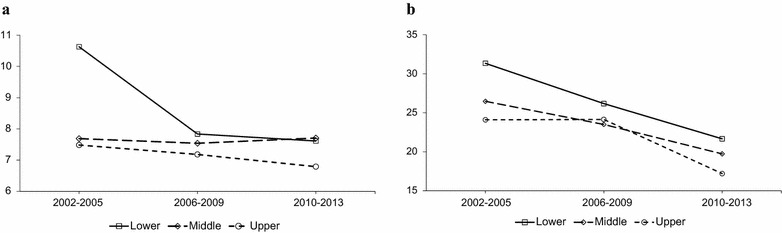
Age-adjusted incidences (per 1000 women) of preeclampsia and postpartum hemorrhage according to household income level in the Korean National Health Insurance cohort database, from 2002 to 2013. **a** Preeclampsia treated with MgSO_4_, **b** Postpartum hemorrhage treated with blood transfusion

**Fig. 2:**
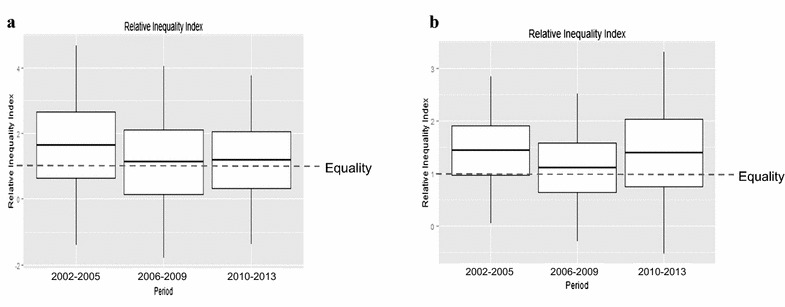
Relative inequality index (RII_KM_) for preeclampsia and postpartum hemorrhage in the Korean National Health Insurance cohort database, from 2002 to 2013. **a** Preeclampsia treated with MgSO_4_ treatment, **b** Postpartum hemorrhage treated with blood transfusion

The incidence of postpartum hemorrhage was generally higher than that of preeclampsia. The incidence rate of preeclampsia was 7.2 in 2002 and 4.7 in 2013. Postpartum hemorrhage occurred in 34.0 cases in 2002, and 20.5 in 2013, per 1000 women. The annual age-adjusted incidence of preeclampsia did not show a significant unidirectional trend (average annual percent change = 0.20, P = 0.941). Postpartum hemorrhage decreased during the study period (average annual percent change = −4.23, P = 0.001; data not shown). The incidence rates of preeclampsia and postpartum hemorrhage in each income group for the three periods are presented in Fig. 1. The incidence of preeclampsia has substantially decreased for the lower income group compared to the other income groups. The incidence of postpartum hemorrhage showed a generally decreasing pattern for the all three income groups. When the absolute and relative inequality measures, which incorporate the population share, were calculated, there was no significant change over the three periods for preeclampsia or postpartum hemorrhage. The relative inequality indices are presented in Fig. 2.

## Discussion

This study demonstrated that lower household income was an independent risk factor for developing preeclampsia and postpartum hemorrhage. During the study period, the inequality indices, considering each income group’s population share, were insignificant for both preeclampsia and postpartum hemorrhage. Despite the global economic recession, there was no evidence of worsening in the absolute or relative disparities in the incidences of preeclampsia or postpartum hemorrhage in Korea. Our study showed both preeclampsia and postpartum hemorrhage would be more prevalent in lower economic status populations, consistent with previous reports (Haelterman et al. 2003; Lindquist et al. 2013, 2014). To the best of our knowledge, this is the first reported study on temporal changes in income-based disparities in preeclampsia and postpartum hemorrhage using population-based data.

Given the widening gap in overall health between the more and less deprived groups in many countries (Son et al. 2012; Bacigalupe and Escolar-Pujolar 2014; Katikireddi et al. 2012), the finding of no significant disparities in preeclampsia and postpartum hemorrhage suggests that the presentation of disparities can differ, depending on health outcome and socioeconomic classification. According to Braveman et al. (2001) an income-based disparity was observed for lower birth weight and delayed antenatal visits, but not for unintended pregnancy. In a US study, the risks of low birth weight and preeclampsia we**r**e higher in African-Americans than in Hispanics while gestational diabetes was more prevalent in Hispanics than in African-Americans (Brown et al. 2007). A study in the UK on temporal trends in adverse perinatal outcome according to socioeconomic disparities showed different patterns of disparities between preterm birth and low birth weight (Glinianaia et al. 2013). In a research on female Spanish population, maternal education gradients in adverse perinatal outcome were significant only for the period 2009–2011, suggesting a negative impact of the economic recession (Juarez et al. 2014). To confirm the findings of this study, studies assessing the gradients of different socioeconomic factors, such as education, in other maternal complications would be needed.

The decreasing tendency of postpartum hemorrhage seen in both the lower and higher income groups could be attributable to several factors. Because essential antenatal services have been covered by the NHI since the late 1990s in Korea, utilization of the services has increased. With improved universal access to care, the frequency of antenatal visits is likely to rise, especially in the lower income group (Ahmed and Khan 2011). However, as the ‘inverse equity hypothesis’ argues, an increase in visits also occurs in women belonging to the higher household income group, because those with a higher socioeconomic status tend to respond to public health initiatives more quickly (Victora et al. 2000). More frequent perinatal visits in these two income groups could have resulted in more—and earlier—detection and management of obstetric hemorrhage, preventing severe hemorrhages requiring blood transfusions (Kim and Moon 2014). The difference in the trend of postpartum hemorrhage underscores the need for monitoring of maternal health status according to household income level.

It is unclear why lower economic status would lead to a higher risk of obstetric complications (Murray et al. 1998; Maharaj 2007). A study of Scottish women showed that the risk of bleeding during pregnancy was higher in lower social class women (Bhandari et al. 2014). Because they are more likely to have an underlying iron deficiency, a certain amount of bleeding could be more risky for women in deprived groups (Bodnar et al. 2002). Furthermore, for a similar reason, emergency hysterectomies were more frequent in women with a lower socioeconomic status (Chestnut et al. 1985). Lower household income is closely linked with a lower level of education, unhealthy behaviors, a more stressful environment, limited access to adequate housing or utilities, increased maternal infection, and unwanted pregnancies, resulting in a lower commitment to prenatal care (Raisanen et al. 2014b). A study in Australia indicated that women belonging to the lowest socioeconomic group generally reported a poorer experience of care during pregnancy, while also having a higher risk of hospital admission or transfer during labor and delivery, in addition to being less likely to have had any antenatal care or postnatal visits (Yelland et al. 2012). The combined linkage among the low socioeconomic status and obstetric risk factors with maternal complication would be a subject of future study.

Several weaknesses due to the use of ICD-10 codes for case definition were unavoidable in this study. The lack of information on disease severity, a possible tendency toward over-reporting by physicians, and misclassification or miscoding might have affected the result of this study. The level and location (urban or rural area) of individual institutions might also have influenced the diagnosis and treatment of preeclampsia or postpartum hemorrhage. However, by using a stratified random sample and strict working definitions, these weaknesses could have been minimized. Validation of the presently used working definitions of preeclampsia and postpartum hemorrhage in the NHI dataset will be a subject of future studies.

Another limitation of this study was that mediating variables, such as healthcare utilization and disease severity, were not considered in the model. Because this study included cases covered by the diagnosis-related groups (DRG)-based payment system, the number of perinatal visits or length of stay could not be used in the analysis. In addition, preeclampsia generally resolves after delivery and the length of hospital stay can thereby be determined by gestational age. Due to the lack of information on gestational age, disease severity could not be evaluated by the length of hospital stay. As only severe cases that needed treatment (MgSO_4_ or blood transfusion) were included in the study, an income-based disparity in the mild form of disease would confirm the findings of this study.

In summary, a lower level of household income was an independent risk factor for developing preeclampsia and postpartum hemorrhage. For both complications, trends in occurrence differed according to household income.

## Conclusions

Our findings suggest that income-based disparities in preeclampsia and postpartum hemorrhage may have been only minimally affected in Korea by the economic recession. Universal coverage of essential maternal care services and attempts to monitor and reduce disparities in health across different socioeconomic groups will be critical for maintaining equity in maternal health.
